# The prevalence and risk factors associated with obesity among adults in Hawtat Bani Tamim: a cross-sectional study

**DOI:** 10.3389/fpubh.2026.1877282

**Published:** 2026-10-22

**Authors:** Mohammed Omar Musa Mohammed

**Affiliations:** College of Business Administration in Hawtat Bani Tamim, Prince Sattam Bin Abdulaziz University, Al-Kharj, Saudi Arabia

**Keywords:** adult, body mass index, logistic regression, obesity, prevalence, risk factors

## Abstract

**Background:**

Obesity has emerged as a significant public health issue in Saudi Arabia, showing regional variations in its prevalence and associated risk factors. This study provides the first city-level estimates of obesity prevalence and its independent sociodemographic and lifestyle determinants in Hawtat Bani Tamim, addressing the gap in localized epidemiological data for targeted public health interventions.

**Methods:**

A cross-sectional study was conducted with 384 adults living in Hawtat Bani Tamim. Sociodemographic, lifestyle, and clinical data were collected through standardized questionnaires and anthropometric measurements. Multivariate logistic regression was used to identify factors independently associated with obesity.

**Results:**

The prevalence of obesity in the study population was 28.9%. Increased odds of obesity were observed in individuals aged 41–50 years (OR = 1.45, *p* = 0.001) and 51–60 years (OR = 2.40, *p* < 0.001), males (OR = 1.20, *p* = 0.007), those with lower education levels (secondary school or less, OR = 4.18, *p* = 0.008), short sleep duration (OR = 1.25, *p* = 0.021), smokers (OR = 1.30, *p* = 0.008), physically inactive individuals (OR = 1.60, *p* = 0.005), and those with hypertension. Moderate activity was protective (OR = 0.75, *p* = 0.028). There is no significant association between obesity and place of residence, marital status, occupation, or most dietary factors.

**Conclusion:**

Obesity is prevalent among adults in Hawtat Bani Tamim and is closely linked to age, gender, education level, sleep hours, smoking, physical inactivity, and hypertension. These results highlight the importance of targeted public health efforts and policies to reduce obesity rates and prevent related health issues in this area.

## 1 Introduction

Obesity, defined as an abnormal or excessive accumulation of body fat that may impair health, affects a growing proportion of adults worldwide. According to the World Health Organization (WHO), obesity in adults is defined as a body mass index (BMI) of ≥30 kg/m^2^ (1). Its prevalence varies across populations and regions, reflecting differences in sociodemographic characteristics, lifestyle patterns, and methods of measurement. In Saudi Arabia, obesity represents a major public health concern. National and regional surveys have reported obesity prevalence estimates ranging from 24.7 to 39.5% across different regions (2–4). More recent national statistics from the General Authority for Statistics (GASTAT) indicate that 23.1% of adults aged 15 years and older were living with obesity in 2024 (5, 6). Similarly, recent evidence suggests that obesity prevalence among Saudi adults has remained relatively stable, ranging from 21.4 to 22.2% between 2020 and 2023 (7). In the Makkah region, obesity prevalence among adults has also been reported to be approximately 23%, broadly consistent with national estimates (8).

Obesity in Saudi Arabia is influenced by multiple factors, including physical inactivity, dietary behaviors, and sociodemographic characteristics (9–14). Although these factors have been examined at the national and regional levels, evidence from smaller cities and rural communities remains limited. Little is known about the prevalence and determinants of obesity among adults in Hawtat Bani Tamim governorate. This gap is important because patterns of obesity and its associated factors may differ across communities according to local socioeconomic conditions, dietary practices, physical activity patterns, and access to health services. Generating community-level evidence can therefore help identify locally relevant risk factors and inform public health strategies that are better adapted to the needs of the population.

Understanding the local burden of obesity is also relevant to broader national and global health priorities. In Saudi Arabia, where overweight and obesity affect a substantial proportion of the adult population, identifying modifiable risk factors may support more targeted prevention efforts and contribute to the objectives of Saudi Vision 2030. At the global level, such efforts are aligned with the Sustainable Development Goals, particularly SDG 3 (Good Health and Wellbeing), which emphasizes the prevention and management of noncommunicable diseases and the promotion of population health. Local epidemiological data can contribute to these goals by providing a basis for evidence-informed interventions, resource allocation, and monitoring of progress.

Therefore, this study aimed to estimate the prevalence of obesity and identify independent sociodemographic, lifestyle, and health-related factors associated with obesity among adults in Hawtat Bani Tamim governorate, Saudi Arabia, using survey-adjusted multivariable logistic regression.

## 2 Materials and methods

### 2.1 Study design and setting

A community-based cross-sectional study was conducted among adults in the Hawtat Bani Tamim governorate, Riyadh Region, Saudi Arabia, from September to October 2025. This period corresponds to moderate spring conditions in the region, with temperatures ranging from 18 to 32 °C. These conditions minimize seasonal influences on physical activity and dietary patterns by enabling outdoor activities without the extreme heat of summer or the cold of winter.

### 2.2 Study population and sampling

The study population comprised men and women aged 18 years or older who had lived in Hawtat Bani Tamim for at least 6 months. The governorate has an estimated population of 41,854 (2022). Eligible participants were required to provide informed consent and undergo anthropometric measurements. Exclusion criteria included pregnancy, bedridden status, physical disabilities that would hinder accurate measurements, temporary residency, and severe cognitive or psychiatric impairments that would compromise data quality or participant safety.

A stratified multistage cluster sampling design was used to enhance representativeness and reduce selection bias. The sampling procedure involved four stages (Table 1):

**Table 1:** Sampling stages of the stratified multistage cluster design.

Stage	Description	Selection method	Unite selected
Stratification	Division into four administrative sub-districts	Proportional to population size	4 strata
Cluster selection	Selection of residential clusters/blocks within strata	Probability-proportional-to-size (PPS)	30 clusters
Household selection	Sampling of households within each cluster	Systematic random sampling	12 households per cluster
Participant selection	Eligible adults within selected households	Simple random sampling	1 adult per household (*n* = 384 targeted)

*Stage 1: Stratification*. Hawtat Bani Tamim was divided into four administrative sub-districts (strata) proportional to population size.

*Stage 2: Cluster selection*. Thirty residential clusters or blocks were selected from within each stratum using probability-proportional-to-size (PPS) sampling.

*Stage 3: Household selection*. Twelve households were systematically sampled from each selected cluster using systematic random sampling.

*Stage 4: Participant selection*. One eligible adult per household was selected via simple random sampling to minimize selection bias and ensure all eligible adults had an equal probability of participation.This detailed sampling strategy ensured that the final sample accurately represented the governorate’s diverse population at the administrative, residential, household, and individual levels.

The sample size was calculated using the standard formula for proportion estimation:

$$n=\frac{Z^{2}\times P\times (1-P)}{E^{2}}$$

where Z is the Z-score for a 95% confidence level (1.96), P is the estimated proportion (0.5 for maximum variability), and E is the margin of error (0.05). Substituting these values yields a minimum sample size of 384 participants.

The proportion P=0.5 was chosen rather than using prevalence estimates from national data because this value maximizes the required sample size and provides the most conservative estimate. When there is uncertainty or variability in the prevalence of the outcome, using P=0.5 is a standard statistical approach that prevents underestimating the required sample size and improves the study’s representativeness and accuracy. National prevalence data were not used because estimates can vary by region and may not accurately reflect the specific study population in Hawtat Bani Tamim. Accordingly, 384 questionnaires were distributed to selected adult participants, and all were completed.

Given the multistage cluster sampling design, the potential impact of the design effect (Deff) on variance estimates was considered. Although the final sample size was 384, the initial sample size calculation was based on simple random sampling assumptions because no prior estimates of the intra-cluster correlation coefficient (ICC) were available for the study setting. To account for clustering in the analysis, design-based methods were used, including sampling weights and cluster-adjusted variance estimation, yielding robust standard errors and valid confidence intervals.

### 2.3 WHO questionnaire and pilot testing

Data were collected using the World Health Organization (WHO) STEPS Approach to Noncommunicable Disease Risk Factor Surveillance (WHO STEPS) questionnaire (Version 3.2). The questionnaire comprised four domains: sociodemographic characteristics, behavioral risk factors (including tobacco use, physical activity, and diet), medical history, and physical measurements. The official Arabic version of the questionnaire was used to ensure linguistic and cultural appropriateness for the study population. The questionnaire was pilot-tested before data collection to assess clarity and feasibility, and minor contextual modifications were made without altering the instrument’s original content. The complete questionnaire is provided as Supplementary material.

### 2.4 Anthropometric measurements and definition of obesity

Trained data collectors obtained anthropometric measurements following the standardized WHO STEPS protocol. Body weight was measured to the nearest 0.1 kg using a calibrated digital scale (Seca 874) with participants wearing light clothing and no shoes. Standing height was measured to the nearest 0.1 cm using a portable stadiometer (Seca 213), with participants standing erect, heels together, and head positioned in the Frankfurt plane. Body mass index (BMI) was calculated as weight (kg) divided by height squared (m^2^) and classified according to WHO criteria. The primary outcome variable was obesity, defined as a binary variable (1 = participants with obesity, 0 = participants without obesity) based on WHO BMI cutoffs. Data were entered into a secure electronic database and checked for completeness and consistency.

### 2.5 Covariates and other variables

Several sociodemographic, behavioral, and lifestyle factors were examined as independent predictors. Gender was coded as male = 1 and female = 2. Age was grouped into five categories: 20–30, 31–40, 41–50, 51–60, and over 60 years. Education level was categorized as secondary school or less (1), university (2), and higher education (3). The place of residence was defined as rural = 1 and urban = 2. Occupation was divided into students = 1, government employee = 2, private-sector employee = 3, and freelance = 4. Marital status was recorded as married = 1 and single = 2. Smoking was coded as no = 0 and yes = 1. Hypertension was assessed during the survey using standardized blood pressure measurements in accordance with the WHO STEPS protocol. Participants with a systolic blood pressure ≥140 mmHg, a diastolic blood pressure ≥90 mmHg, or who were currently taking antihypertensive medication were classified as having hypertension. The questionnaire did not include an item assessing previous physician-diagnosed hypertension; therefore, self-reported history of hypertension was not used for classification. Physical activity was categorized as inactive, low, moderate, or high based on the reported frequency of exercise per week, type of physical activity, and typical duration of each session. Respondents who reported no regular exercise were classified as inactive; those exercising 1–2 days per week for less than 30 min per session were classified as low; those exercising 3–4 days per week for 30–59 min per session were classified as moderate; and those exercising 5 or more days per week for at least 60 min per session were classified as high. Sleeping hours per day were categorized as short sleeping (3–5 h), standard sleeping (6–9 h), and long sleeping (9 h or more). Vegetable daily consumption was categorized by the number of servings consumed per day as never (0 servings), low (1–2 servings), moderate (3–4 servings), and high (5 or more servings). These variables have been utilized in numerous prior studies (15–19).

### 2.6 Statistical analysis

Statistical analyses were conducted using SAS version 9.4 M9 (SAS Institute Inc., Cary, NC, USA). Descriptive statistics were used to summarize participants’ characteristics and obesity prevalence. Differences in obesity prevalence across categorical variables were assessed using the Pearson chi-square test. For variables with more than two categories, the overall chi-square test was used to assess statistical significance. A *p*-value <0.05 was considered statistically significant.

Survey-weighted binary logistic regression was used to examine the associations between obesity (yes/no) and selected sociodemographic, lifestyle, and health-related factors (20–24). Covariates were selected *a priori* based on epidemiological relevance and previous literature and were entered simultaneously into the multivariable model. Model parameters were estimated using maximum likelihood estimation (25–29).

Adjusted odds ratios (AORs) with corresponding 95% confidence intervals (CIs) were reported. Categorical variables were entered using indicator (dummy) coding with appropriate reference categories.

All descriptive and regression analyses accounted for the complex survey design by incorporating sampling weights, clustering, and stratification. Sampling weights, calculated as the inverse probability of selection and adjusted for non-response, were applied to obtain population-representative estimates. Multicollinearity among covariates was assessed using variance inflation factors (VIFs), and model adequacy was evaluated using appropriate goodness-of-fit and diagnostic procedures (28, 30–36). All statistical tests were two-sided, and a *p*-value <0.05 was considered statistically significant.

### 2.7 Ethical considerations

This research adhered to the Declaration of Helsinki guidelines. Ethical approval for this study was obtained from the Deanship of Research at Prince Sattam University (SCBR-5222025, 24 August 2025). Informed consent was obtained from all participants before their inclusion in the study, ensuring they were fully aware of the study’s purpose, procedures, risks, and benefits. Participants who declined participation or withdrew consent were classified as non-respondents. No replacements were provided for non-responding individuals or households to preserve the integrity of the sampling design. The overall response rate was calculated based on all eligible participants who completed the survey.

## 3 Results

A total of 420 eligible adults were approached to participate in the study. Of these, 36 declined to participate or were excluded for incomplete data, leaving a final analytical sample of 384 participants. The findings are presented according to the study objectives.

Table 2 presents the characteristics of the participants. 28.9% of the participants were classified as having obesity, while 71.1% were not. Females comprised the majority (59.6%), while males accounted for 40.4%. The age distribution indicated that most participants were young to middle-aged adults: 24.5% were aged 20–30, 24.7% were aged 31–40, and 22.7% were aged 51–60, whereas only 10.7% were aged 60 or older. The majority had a university-level education (71.1%), followed by those with secondary or lower education (16.7%) and those with higher education (12.2%). A larger proportion of the sample lived in urban areas (68.7%) than in rural areas (31.3%). Regarding occupation, nearly half (48.2%) were government employees, 14.6% worked in the private sector, 28.4% were freelancers, and 8.9% were students. Regarding marital status, most were single (69%), while 31% were married.

**Table 2:** Characteristics of the participants.

Characteristic	Frequency	Percentage
Obesity		
No	273	71.1
Yes	111	28.9
Gender		
Male	155	40.4
Female	229	59.6
Age (in years)		
20–30	94	24.5
31–40	95	24.7
41–50	67	17.4
51–60	87	22.7
More than 60	41	10.7
Level of education		
Secondary school or less	64	16.7
University	273	71.1
Higher education	47	12.2
Place of residence		
Rural	120	31.3
Urban	264	68.7
Occupation		
Student	34	8.9
Government employee	185	48.2
Private sector employee	56	14.6
Freelance work	109	28.4
Marital status		
Married	119	31
Single	265	69
Smoking		
No	341	88.8
Yes	43	11.2
Hypertension		
No	286	74.5
Yes	98	25.5
Physical activities per day		
Inactive	62	16.1
Low	110	28.6
Moderate	200	52.1
High	12	3.1
Sleep hours per day		
Short sleep	206	53.6
Standard sleep	174	45.3
Long sleep	4	1
Vegetables served per day		
Never	82	21.4
Low	69	18
Moderate	148	38.5
High	85	22.1

Lifestyle and health-related characteristics indicated that most participants were non-smokers (88.8%). The prevalence of hypertension was 25.5% among the study participants. Regarding physical activity, over half (52.1%) engaged in moderate activity, 28.6% reported low activity, and a small proportion (3.1%) reported high activity. Sleep duration varied: 53.6% reported short sleep, 45.3% reported regular sleep, and 1% reported long sleep. Regarding vegetable consumption, most participants reported moderate (38.5%) or high (22.1%) intake, although 21.4% reported never consuming vegetables.

Table 3 presents the prevalence of obesity across categories of participant characteristics, together with the corresponding Pearson chi-square *p*-values. The prevalence of obesity did not differ significantly between males and females (29.7% vs. 28.4%, *p* = 0.784). Significant differences in obesity prevalence were observed across age groups (*p* = 0.013), with the highest prevalence among participants aged 41–50 years (37.3%) and 20–30 years (34.0%), and the lowest among those aged 51–60 years (20.7%). Obesity prevalence did not differ significantly by place of residence (rural vs. urban; *p* = 0.419) or occupation (*p* = 0.383).

**Table 3:** Prevalence of obesity according to participants’ sociodemographic and biological characteristics.

Characteristics	Obesity status		Overall *p*-value
	No (%)	Yes (%)	
Gender			
Male	109 (70.3%)	46 (29.7%)	0.784
Female	164 (71.6%)	65 (28.4%)	
Age (in years)			
20–30	62 (66%)	32 (34%)	0.013
31–40	71 (74.7%)	24 (25.3%)	
41–50	42 (62.7)	25 (37.3%)	
50–60	69 (79.3%)	18 (20.7%)	
More than 60	29 (70.7%)	12 (29.3%)	
Place of residence			
Rural	84 (70%)	36 (30%)	0.419
urban	189 (71.6%)	75 (28.4%)	
Level of education			
Secondary school or less	39 (60.9%)	25 (39.1%)	0.04
University	195 (71.4%)	78 (28.6%)	
Higher education	39 (83%)	8 (17%)	
Occupation			
Student	26 (76.5%)	8 (23.5%)	0.383
Government employee	127 (68.6%)	58 (31.4%)	
Private sector employee	37 (66.1%)	19 (33.9%)	
Freelance work	83 (76.1%)	26 (23.9%)	
Marital status			
Married	78 (65.5%)	41 (34.5%)	0.041
Single	195 (73.6%)	70 (26.4%)	
Smoking			
No	241 (70.7%)	100 (29.3%)	0.037
Yes	32 (74.4%)	11 (25.6%)	
Hypertension			
No	217 (75.9%)	69 (24.1%)	< 0.001
Yes	56 (57.1%)	42 (42.9%)	
Physical activities per day			
Inactive	37 (59.7%)	25 (40.3%)	0.002
Low	91 (82.7%)	19 (17.3%)	
Moderate	134 (67%)	66 (33%)	
High	11 (91.7%)	1 (8.3%)	
Sleep hours per day			
Short sleep	141 (68.4%)	65 (31.6%)	<0.001
Standard sleep	129 (74.1%)	45 (25.9%)	
Long sleep	3 (75%)	1 (25%)	
Vegetables served per day.			
Never	59 (72%)	23 (28%)	0.028
Low	54 (78.3%)	15 (21.7%)	
Moderate	93 (62.8%)	55 (37.2%)	
High	67 (78.8%)	18 (21.2%)	

Obesity prevalence differed significantly across educational levels (*p* = 0.040), with the highest prevalence among participants with secondary education or less (39.1%) and the lowest among those with higher education (17.0%). A significant difference was also observed across marital-status categories (*p* = 0.041), with a higher prevalence among married participants than among single participants (34.5% vs. 26.4%). Obesity prevalence differed significantly by smoking status (*p* = 0.037), with a higher prevalence among non-smokers than smokers (29.3% vs. 25.6%). Participants with hypertension had a higher prevalence of obesity than those without hypertension (42.9% vs. 24.1%, *p* < 0.001).

Significant differences in obesity prevalence were also observed across physical activity categories (*p* = 0.002), with the highest prevalence among inactive participants (40.3%) and the lowest among those with high physical activity (8.3%). Obesity prevalence differed significantly across sleep-duration categories (*p* < 0.001), with a higher prevalence among short sleepers (31.6%) than among participants with standard or long sleep durations. Finally, obesity prevalence differed significantly across vegetable-intake categories (*p* = 0.028), with the highest prevalence among moderate consumers (37.2%).

Table 4 presents results from a survey-weighted multivariable logistic regression examining factors associated with obesity among adults in Hawtat Bani Tamim. Adjusted odds ratios (ORs) and 95% confidence intervals (CIs) are reported for each covariate’s association with obesity, with adjustment for other variables in the model. All covariates were selected *a priori* based on epidemiological relevance and the previous literature. The analysis accounted for the complex survey design by incorporating sampling weights, clustering, and stratification. Statistical significance was defined as *p* < 0.05. The reference category for each categorical variable is indicated as “Ref.” in the table.

**Table 4:** Multivariate logistic regression analysis of factors associated with obesity among adults in Hawtat Bani Tamim (*n* = 384).

Characteristics	OR	95% CI	P–value
Age (in years)			
20–30	0.85	0.70–1.02	0.081
31–40	1.10	0.95–1.30	0.200
41–50	1.45	1.23–1.71	0.001
51–60	2.40	2.05–2.82	<0.001
More than 60	Ref		
Gender			
Male	1.20	1.05–1.37	0.007
Female	Ref		
Place of residence			
Rural	0.83	0.40–1.74	0.622
Urban	Ref		
Marital status			
Single	1.28	0.74–2.23	0.377
Married	Ref		
Occupation			
Student	1.03	0.37–2.88	0.961
Government employee	1.51	0.81–2.82	0.191
Private sector employee	1.69	0.74–3.83	0.212
Freelance work	Ref		
Level of education			
Secondary school or less	4.18	1.44–12.10	0.008
University	2.81	1.08–7.33	0.035
Higher education	Ref		
Sleep hours per day			
Short sleep	1.25	1.03–1.50	0.021
Standard sleep	1.05	0.91–1.22	0.462
Long sleep	Ref		
Smoking			
Yes	1.30	1.07–1.57	0.008
No	Ref		
Hypertension			
No	0.33	0.18–0.60	<0.001
Yes	Ref		
Physical activities per day			
Low	1.20	0.90–1.60	0.210
Inactive	1.60	1.15–2.23	0.005
Moderate	0.75	0.58–0.97	0.028
High	Ref		
Vegetables served per day			
Low	1.04	0.43–2.50	0.936
Moderate	1.02	0.46–2.28	0.953
High	2.34	1.19–4.60	0.013
Never	Ref		

In the survey-weighted multivariable logistic regression, age was significantly associated with obesity. Compared with participants aged 60 years or older, those aged 41–50 years and 51–60 years had higher odds of obesity (OR = 1.45, *p* = 0.001 and OR = 2.40, *p* < 0.001, respectively). Male participants also had higher odds of obesity than female participants (OR = 1.20, *p* = 0.007).

After adjusting for potential confounders, educational level remained significantly associated with obesity. Participants with secondary education or less and those with a university degree had higher odds of obesity than those with higher education (OR = 4.18, *p* = 0.008 and OR = 2.81, *p* = 0.035, respectively). Short sleep duration was associated with higher odds of obesity (OR = 1.25, *p* = 0.021), as was smoking (OR = 1.30, *p* = 0.008). Participants without hypertension had lower odds of obesity than those with hypertension (OR = 0.33, *p* < 0.001). Physical activity also showed significant differences in the odds of obesity: inactive participants had higher odds (OR = 1.60, *p* = 0.005), whereas those with moderate physical activity had lower odds (OR = 0.75, *p* = 0.028). Place of residence, marital status, occupation, and vegetable consumption were not significantly associated with obesity after adjustment.

## 4 Discussion

This study aimed to estimate the prevalence of obesity among adults in Hawtat Bani Tamim, Saudi Arabia, and to identify its socioeconomic, behavioral, and biological correlates. Results revealed a prevalence of 28.9%, underscoring obesity as a key public health issue. Adjusted analysis showed that age, education, physical activity, smoking, sleep duration, and hypertension were significantly associated with obesity, whereas other factors were not independently linked after accounting for confounders. These results can inform the development of targeted public health initiatives and obesity prevention plans in Saudi Arabia.

The present study found that the prevalence of obesity among adults in Hawtat Bani Tamim was 28.9%, underscoring that obesity remains a substantial public health concern in this community. This estimate aligns with reports from other regions of Saudi Arabia, where adult obesity prevalence has ranged from approximately 20 to 40%, depending on the study population and geographic setting. The observed prevalence is also comparable to estimates reported in several Gulf Cooperation Council countries but remains higher than the global average reported by the World Health Organization. These similarities may reflect shared lifestyle and environmental factors, including reduced physical activity, increased consumption of energy-dense foods, urbanization, and socioeconomic transitions. Differences between studies may be attributable to variations in study populations, sampling methods, age distributions, obesity definitions, and the timing of data collection. In summary, the 28.9% obesity prevalence observed is concerning and underscores an urgent need for comprehensive efforts that address not only individual behavior change but also broader structural and educational factors to effectively reduce obesity rates and the related burden of non-communicable diseases in the region (37–42).

The present study identified male sex, lower educational level, short sleep duration, smoking, physical inactivity, and hypertension as factors associated with obesity. Although obesity prevalence varied across age groups, the observed pattern did not indicate a consistent increase with age. The associations observed for other factors may reflect differences in lifestyle behaviors, socioeconomic characteristics, and health-related factors within this population (43–45). Although age was significantly associated with obesity in the unadjusted analysis, this association was attenuated after multivariable adjustment, suggesting that other behavioral and health-related factors may partly explain the observed effect. This finding underscores the importance of accounting for potential confounding when interpreting the relationship between age and obesity. The higher prevalence among men is also noteworthy, as many regional studies have reported either higher rates among women or no significant gender differences; this may reflect local sociocultural factors, occupational patterns, or health-behavior differences between men and women (39, 46–48). Contrary to national trends where women have higher obesity rates (33.5% vs. 24.1%), our study found higher odds among men in Hawtat Bani Tamim. For Saudi women, risk factors include socio-cultural restrictions, marital weight gain, and lower education. Men’s risks involve sedentary jobs, snacking, SSBs, and occupational patterns, especially among younger adults. These local patterns highlight the need for gender-specific interventions tailored to the socio-economic context of Hawtat Bani Tamim. Although sex was not significantly associated with the outcome in the bivariate analysis (*p* = 0.784), it became a significant predictor in the multivariable model (OR = 1.20). This change suggests the presence of confounding effects, whereby adjustment for other covariates revealed the independent association between male sex and the outcome.

Educational attainment proved to be a key risk factor, with lower education levels significantly raising the chances of obesity; this finding aligns well with broader research evidence (40, 49, 50). This inverse relationship could reflect limited health literacy, lower awareness of healthy behaviors, and differential access to resources for healthy living among less educated individuals. Public health interventions may need to target these groups specifically to address knowledge and empowerment gaps regarding obesity prevention.

The positive link between short sleep duration and obesity aligns with growing evidence connecting sleep deprivation to metabolic issues and increased appetite (51, 52). Short sleep mainly contributes to obesity by altering hormonal and metabolic signals that increase hunger and promote fat storage. It increases ghrelin (the hunger hormone), decreases leptin (the satiety hormone), disrupts glucose metabolism, reduces insulin sensitivity, and elevates cortisol, all of which promote weight gain, particularly around the abdomen. Lack of sleep results in fatigue, lowers activity levels, and increases cravings for high-calorie foods. Overall, regularly getting little sleep significantly raises the risk of obesity by affecting energy intake and expenditure.

The current study showed a significant link between smoking and obesity; many studies support this finding (53, 54). This suggests a complex yet consistent connection between smoking and a higher risk of obesity. However, smoking is often associated with lower body weight due to nicotine’s appetite-suppressing effects; heavy tobacco use and quitting smoking have been linked to increased obesity rates and fat accumulation around the abdomen. Many studies report that heavy smokers tend to have higher body weight and BMI compared to light smokers, and former smokers have a higher prevalence of obesity than current or never smokers. These findings are consistent across different populations and emphasize smoking’s dual role in weight regulation, initially reducing weight but leading to insulin resistance and greater central fat accumulation over time. The link between smoking and obesity needs careful interpretation. Smoking was significantly associated with obesity in adjusted analyses, whereas bivariate results were inconsistent, highlighting a complex, possibly bidirectional relationship. Smoking status may reflect behavioral or metabolic differences, such as weight gain after quitting, which this cross-sectional study could not fully disentangle.

Interestingly, higher vegetable consumption was positively associated with obesity in the present study, consistent with findings reported in previous studies (55, 56). However, this unexpected association should be interpreted cautiously. Given the cross-sectional design, temporal relationships cannot be established, and reverse causality is possible; participants with obesity may have increased their vegetable intake as part of efforts to improve their diet or manage their weight. In addition, dietary intake and other behavioral characteristics were self-reported and may be subject to recall and social desirability bias. Similar sources of bias may have influenced other behavioral associations observed in this study. Therefore, this finding should not be interpreted as evidence that higher vegetable consumption causes obesity, but rather as an association that warrants further investigation using longitudinal studies and more detailed assessments of dietary and lifestyle behaviors.

Furthermore, the study found a significant association between physical inactivity and obesity, consistent with findings from previous studies (57–60). The lower prevalence of obesity observed among participants reporting moderate physical activity is also broadly consistent with evidence linking physical activity with lower obesity risk. However, the finding that moderate activity appeared more strongly associated with lower obesity prevalence than high activity warrants cautious interpretation. This pattern may reflect self-reporting bias, misclassification of physical activity levels, differences in the number of participants across activity categories, or other unmeasured factors, including potential compensatory dietary behaviors among highly active individuals. Given the cross-sectional design, these findings should be interpreted as associations rather than causal effects. Nevertheless, they support the importance of community-level strategies to facilitate regular physical activity across different age groups.

The present study identified a significant positive association between obesity and hypertension, consistent with evidence from previous studies (61–64). Previous research has reported substantially higher odds of hypertension among individuals with obesity, with pooled estimates in some studies ranging from approximately 3 to 4. The observed association highlights the importance of considering obesity and hypertension together in cardiovascular risk assessment and prevention. However, given the cross-sectional design of the present study, the temporal direction and causality of this association cannot be established.

The absence of statistically significant associations between obesity and place of residence, marital status, occupation, and most dietary factors in the present study contrasts with findings from previous research, in which these characteristics have been associated with obesity (55, 65–67). Higher obesity prevalence has been reported among urban residents, married individuals, and certain occupational groups, while unhealthy dietary patterns have also been associated with obesity. Conversely, some studies have reported inverse associations between vegetable intake and obesity. The differences between these findings and those of the present study may reflect variation in population characteristics, socioeconomic and environmental contexts, sample size, measurement methods, or the distribution of these factors within the study population. In addition, residual confounding and self-reported measurement of dietary and behavioral variables may have influenced the observed associations. Therefore, the absence of statistically significant associations in this study should not necessarily be interpreted as evidence that these factors have no relationship with obesity.

The findings of this study are relevant to several SDGs, particularly SDG 3 (Good Health and Wellbeing) and, more broadly, SDG 2 (Zero Hunger), which includes addressing malnutrition in all its forms. Identifying factors associated with obesity, including physical inactivity, lower educational attainment, and selected lifestyle behaviors, provides local evidence that may help inform health-promotion and obesity-prevention strategies. These results are also relevant to Saudi Arabia’s Vision 2030, which emphasizes improving population health, promoting healthy lifestyles, and reducing the burden of noncommunicable diseases. Community-level efforts to promote healthy dietary practices, increase opportunities for physical activity, and improve health education may contribute to these broader national and global health objectives. However, because this study is cross-sectional, the identified associations should not be interpreted as causal relationships. Further longitudinal and intervention research is needed to evaluate the effectiveness of strategies for preventing and managing obesity in similar communities.

### 4.1 Public health implications and policies

The findings provide useful evidence for public health planning in Hawtat Bani Tamim and similar communities. The observed prevalence of obesity and its adjusted associations with sociodemographic, lifestyle, and health-related factors highlight the importance of strengthening community-based approaches to obesity prevention. Public health programs could prioritize promotion of regular physical activity, healthy dietary practices, adequate sleep, and health education, particularly among population groups with higher observed odds of obesity. Interventions should be culturally appropriate and adapted to local socioeconomic and environmental conditions. Given the observed association between obesity and hypertension, integrated approaches addressing obesity and other cardiometabolic risk factors may also be beneficial. These findings can support local health authorities in planning targeted health-promotion activities and allocating resources, while recognizing that the cross-sectional design does not establish causal relationships.

### 4.2 Limitations of the study

Certain limitations must be recognized. Cross-sectional design prevents determination of cause-and-effect relationships or temporal sequences among obesity and the sociodemographic, lifestyle, and health factors studied. As a result, the associations found should be viewed as correlations rather than evidence of causality. Future longitudinal research is necessary to better understand the temporal dynamics and potential causal pathways involved.

Secondly, certain potentially important variables were not measured, such as genetic predisposition, psychosocial factors, detailed socioeconomic indicators, total caloric intake, and comprehensive dietary patterns. Not including these variables could have caused residual confounding and restricted the ability to understand the multifaceted causes of obesity fully. Moreover, data on physical activity, sleep duration, and diet were obtained through self-report, which may be affected by recall or reporting bias.

Third, even though a multistage cluster sampling design was employed, the sample size was not explicitly increased to account for a design effect due to the lack of reliable prior estimates of the intracluster correlation coefficient. As a result, clustering might have decreased the estimate precision. Nevertheless, the survey-weighted analysis included sampling weights, clustering, and stratification in calculating population parameters and their variances.

Fourth, another limitation is that post-hoc pairwise comparisons were not conducted following statistically significant overall chi-square tests in the bivariate analysis. Consequently, these analyses indicate whether an overall association exists across categories but do not identify which specific groups differ from one another. However, associations between individual categories were examined in the multivariable logistic regression analysis using specified reference categories, with adjusted odds ratios and 95% confidence intervals reported. Future studies could incorporate appropriately adjusted pairwise comparisons, such as the Holm correction, to further characterize differences between individual groups.

Fifth, the association between higher vegetable intake and obesity should be interpreted carefully because total energy intake and overall dietary patterns were not fully considered. Residual confounding might have influenced this result. Additionally, reverse causation could be at play, as individuals with obesity may have changed their diets, such as eating more vegetables. Further research with detailed dietary assessments is necessary to clarify this relationship.

Finally, although anthropometric measurements were used to calculate BMI, detailed documentation of measurement procedures, including equipment calibration, inter-observer reliability, and standardized training of data collectors, was limited. This may have introduced measurement variability and potential misclassification of BMI. Despite these limitations, the study provides context-specific evidence on obesity prevalence and its associated factors in Hawtat Bani Tamim. The findings should be interpreted as associations rather than causal relationships. They can serve as a basis for future longitudinal and community-based studies that incorporate more comprehensive behavioral, dietary, and socioeconomic measures.

### 4.3 Future research directions

Future research in Hawtat Bani Tamim should focus on conducting longitudinal studies to better understand the temporal links between obesity and associated sociodemographic, lifestyle, and health factors. Additionally, intervention studies are essential to assess the success of culturally tailored strategies aimed at preventing and managing obesity, especially among groups with higher obesity odds. Future investigations should utilize objective measures for lifestyle behaviors, such as physical activity and sleep, where possible, and incorporate qualitative methods to explore the contextual barriers and enablers of healthy behaviors.

More comprehensive dietary assessments, such as 24-h dietary recalls or food frequency questionnaires, should be considered to capture total energy intake, macronutrient composition, dietary patterns, and food-preparation practices. This information would help investigate the observed association between vegetable consumption and obesity and enable better adjustment for potential dietary confounding. Repeated community-based surveys could also help monitor changes in obesity prevalence and associated factors over time. Collaboration with local health authorities and relevant sectors could facilitate the development and evaluation of community-based interventions and support the translation of locally generated evidence into public health planning.

## 5 Conclusion

This study found that obesity was prevalent among adults in Hawtat Bani Tamim, with nearly one-third of participants classified as having obesity. In the adjusted analysis, several sociodemographic, lifestyle, and health-related factors were significantly associated with obesity. However, these findings should be interpreted cautiously because of the cross-sectional design, potential residual confounding, and reliance on self-reported measures. The observed associations do not establish causality and should not be generalized beyond the study population without further evidence. The findings provide context-specific evidence that may support local obesity prevention and health-promotion efforts. Longitudinal studies using objective measurements and more comprehensive assessments of dietary and behavioral factors are needed to clarify temporal relationships and further investigate the determinants of obesity in smaller communities in Saudi Arabia.

## Data Availability

The raw data supporting the conclusions of this article will be made available by the authors, without undue reservation.
