# Supplementary material for: The prevalence and risk factors associated with obesity among adults in Hawtat Bani Tamim: a cross-sectional study
Source: Front Public Health. 2026 Oct 22;14:1877282. doi: 10.3389/fpubh.2026.1877282 (PMC13738999; doi:10.3389/fpubh.2026.1877282)
Supplement: Supplementary file 1 [file Table_1.DOCX]

**Questionnaire: The Prevalence and Risk Factors Associated with Obesity Among Adults in Hawtat Bani Tamim**

**Introduction**

You are kindly invited to participate in a research study titled **“The Prevalence and Risk Factors Associated with Obesity Among Adults in Hawtat Bani Tamim: A Cross-Sectional Study.”** This study aims to estimate the prevalence of obesity and examine its associated risk factors among adults residing in Hawtat Bani Tamim. Your participation is voluntary, and you may decide whether to take part.

**Informed consent**

Before proceeding, please read the following information carefully. The questionnaire asks about your age, sex, education, occupation, dietary habits, physical activity, and other health-related factors. Completing the questionnaire will take approximately a few minutes. All information collected will be kept confidential and used solely for research purposes. No names or other identifying details will be requested, and your responses will be reported only in aggregated form.

Your participation is entirely voluntary. You may skip any question you do not wish to answer and may withdraw from the study at any time without any penalty or loss of benefit. There are no known risks beyond the inconvenience of answering the questions.

**Consent statement:**

- I have read and understood the information provided above.
- I am 18 years old or older.
- I voluntarily agree to participate in this study.

[ ] I agree to participate
[ ] I do not agree to participate

Section 1: Demographics

- What is your age?
- What is your gender? (Male/Female)
- What is your marital status? (Single/Married)
- What is your highest level of education? (Secondary school or less/University /Higher education)
- What is your employment status? (Student/Government employee/Private sector employee/Freelance work)
- Do you live in an urban or rural area?

Section 2: Anthropometric Data

- What is your current weight? (kg)
- What is your height? (cm)
- BMI (it was calculated)
- Hypertension (Yes/ No)
- Smoking (Yes/No)

Section 4: Physical Activity per day

- How many days per week do you exercise?
- What types of physical activity do you engage in? (Walking/Running/Gym/Sports/Other)
- How long is your typical exercise session? (Minutes)
- How would you rate your physical activity level? (inactive/Low/Moderate/High)

Section 5: Sleep and Other Lifestyle Factors

- On average, how many hours do you sleep per night?

Section 5: Vegetables served per day

- During a usual day, how many servings of vegetables do you eat (Never/Low/Moderate/High)
